# A case of vasculitis triggered by infective endocarditis in a patient undergoing maintenance hemodialysis: a case report

**DOI:** 10.1186/s12882-021-02647-w

**Published:** 2022-01-03

**Authors:** Hanui Park, Miji Lee, Jin Seon Jeong

**Affiliations:** 1 Department of Internal Medicine, Division of Nephrology, Seoul Sacred Heart General Hospital, Seoul, South Korea; 2 Department of Pathology, Veterans Health Service Medical Center, Seoul, South Korea; 3 Department of Internal Medicine, Division of Nephrology, Veterans Health Service Medical Center, 53 Jinhwangdo-ro 61-gil, Gangdong-gu, Seoul, 05368 South Korea

**Keywords:** IgA vasculitis, Hemodialysis, Infective endocarditis, Leukocytoclastic vasculitis, *Clostridium difficile* infection

## Abstract

**Background:**

Immunoglobulin A vasculitis (IgA vasculitis) is one of the most common forms of vasculitis in children. It rarely occurs in adults. It is a systemic vasculitis with IgA deposition and is characterized by the classical tetrad of purpura, arthritis/arthralgia, gastrointestinal and renal involvement. Certain types of infections, and pharmacological agents have been reported to be associated with IgA vasculitis. Here, we describe a case of IgA vasculitis triggered by infective endocarditis in a patient undergoing maintenance hemodialysis.

**Case presentation:**

A 70-year-old man undergoing hemodialysis was admitted because of skin purpura, abdominal pain, diarrhea, and lower back pain. We suspected him as IgA vasculitis based on the clinical features and skin biopsy findings. Transesophageal echocardiography revealed infective endocarditis, which predisposed him to IgA vasculitis. He was treated with antibiotics and low-dose corticosteroids, which led to resolution of vasculitis.

**Conclusions:**

This is the first case of IgA vasculitis triggered by infective endocarditis in a patient undergoing hemodialysis. Patients undergoing hemodialysis are at a high risk of infection because of immune dysfunction and frequent venipuncture. The incidence of infective endocarditis associated with IgA vasculitis is very low, but it has been repeatedly reported. Therefore, it is necessary to consider infective endocarditis in patients with clinical features that indicate IgA vasculitis.

## Background

Immunoglobulin A (IgA) vasculitis, also known as Henoch–Schönlein purpura, is a systemic small-sized vasculitis with IgA deposition and is characterized by non-thrombocytopenic palpable purpura, arthritis/arthralgia, gastrointestinal involvement, and renal involvement. It is uncommon in adults, with an annual incidence of 8–18 per million, compared with an annual incidence of 30–267 per million in children [1, 2]. IgA vasculitis recovers spontaneously, its treatment is often symptomatic. Supportive management, including bed rest and nonsteroidal anti-inflammatory drug administration, is the main treatment for mild IgA vasculitis. In cases with severe gastrointestinal involvement or rapid renal impairment, steroids and/or immunosuppressive drugs are required [3, 4].

IgA vasculitis rarely occurs in patients on maintenance hemodialysis; only 5 such cases have been reported [5–9]. Although its pathogenesis remains uncertain, predisposing factors such as mucosal infection and use of certain medication have been reported. Infective endocarditis could trigger IgA vasculitis, and a few cases have been reported since 1987 [10]. We present a case of IgA vasculitis associated with infective endocarditis in a patient on maintenance hemodialysis.

## Case presentation

**Fig. 1:**
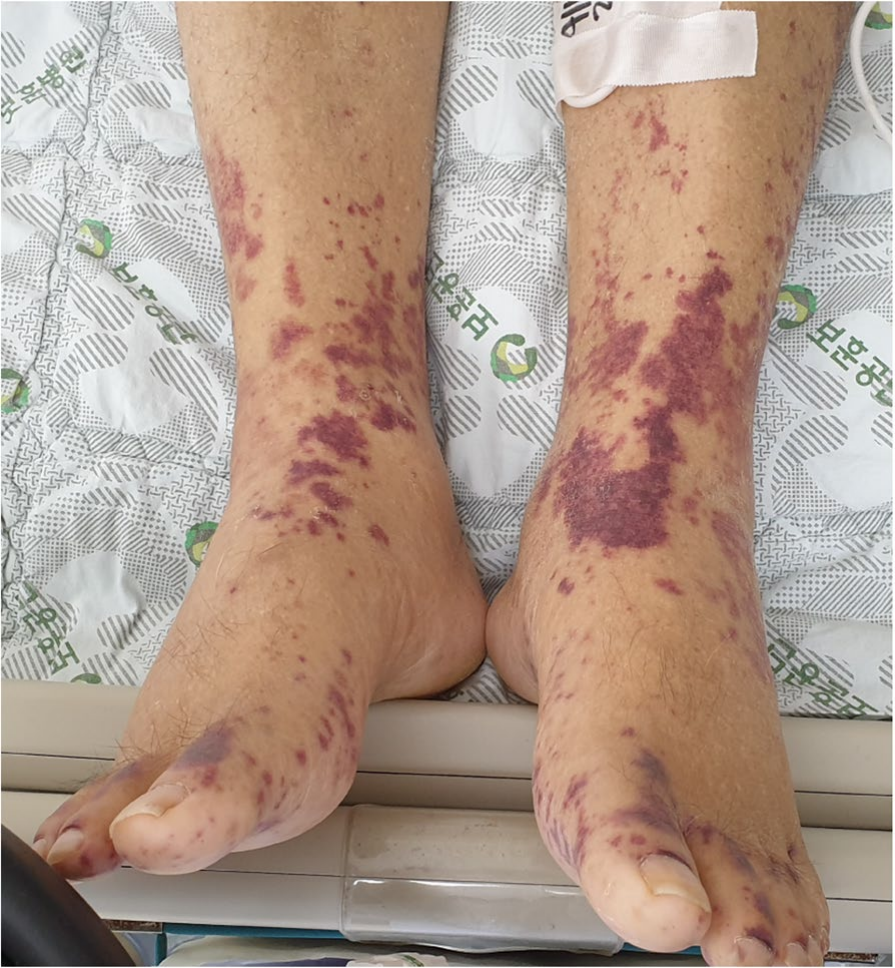
Palpable purpura over the lower extremities

A 70-year-old man with diabetes mellitus and end-stage renal disease (ESRD) presumed to be due to diabetic nephropathy (no kidney biopsy was performed) who was undergoing hemodialysis since 2 years was admitted to the hospital with a 7-day history of lower back pain, watery diarrhea, and bilateral lower leg purpura. One month earlier, he had been admitted for fever, diagnosed with an arteriovenous graft infection, and treated with antibiotics and surgical removal of the graft. His vital signs on admission were as follows: blood pressure, 152/66 mmHg; heart rate, 90 BPM [(beats per minute (BPM)]; and body temperature, 36.6 °C. Physical examination revealed abdominal distension and tenderness without muscle guarding. He had no history of trauma that could cause lower back pain and no evidence of it on spine radiography. Erythematous palpable purpura was present over both lower legs [Fig. 1]. Initial laboratory test results showed the following: leukocytosis with a white blood cell count of 12.66 x 10^12^cells/L (normal: 4.2–5.9 x 10^12^cells/L) with 88.2% neutrophils (normal: 50–75%), C-reactive protein level of 148.86 mg/L (elevated, normal: < 3.0 mg/L), and procalcitonin level of 3.47 ng/dL (elevated, normal: < 0.5 ng/dL). The platelet count and liver function test results were within normal limits. Plain abdominal radiography revealed mild ileus without signs of bowel obstruction.

We suspected colitis and intravenously administered metronidazole. The presence of *Clostridium difficile* (C.difficile) toxins A and B was confirmed on stool examination. *C. difficile* infection was diagnosed based on diarrhea, antibiotic history, and positive toxin test. Oral vancomycin was administered. On day 3 of hospitalization, methicillin-resistant *Staphylococcus aureus* (MRSA) was isolated from blood culture; therefore, and we intravenously administered vancomycin for treating MRSA bacteremia.

**Fig. 2:**
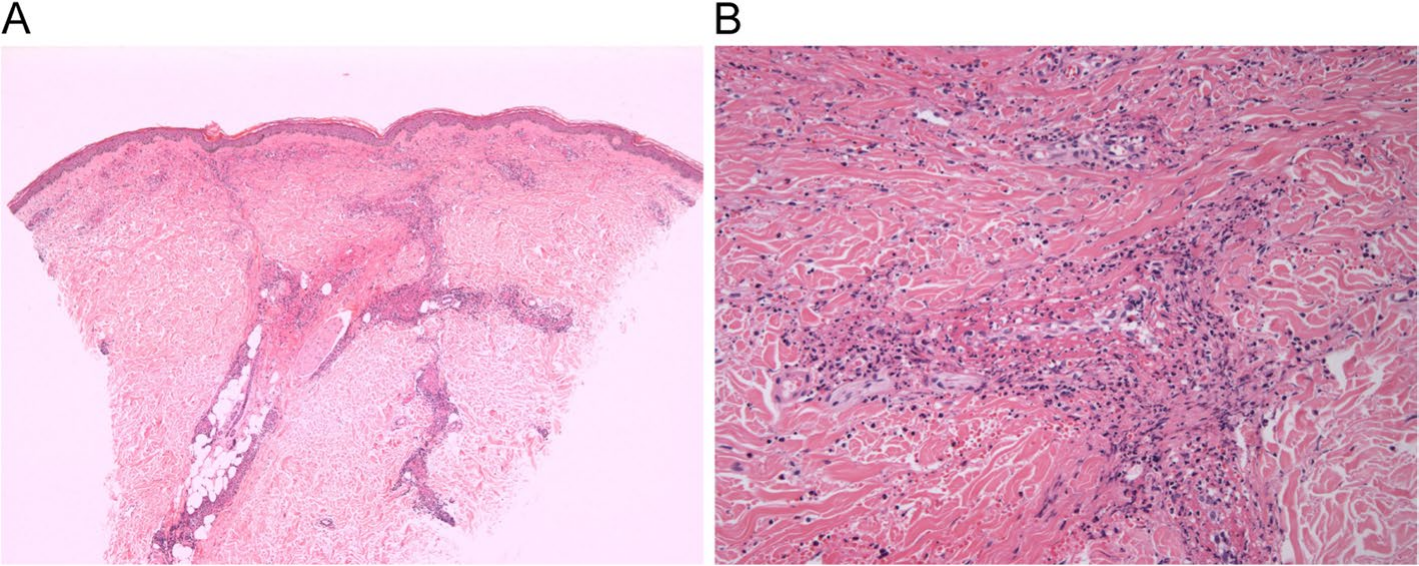
Histologic features of leukocytoclastic vasculitis are observed on skin biopsy. (A) On low power view, perivascular extravasated red blood cells are noted (hematoxylin and eosin, × 40). (B) On high power view, fibrinoid vascular necrosis with nuclear dust is seen (hematoxylin and eosin, × 200)

**Fig. 3:**
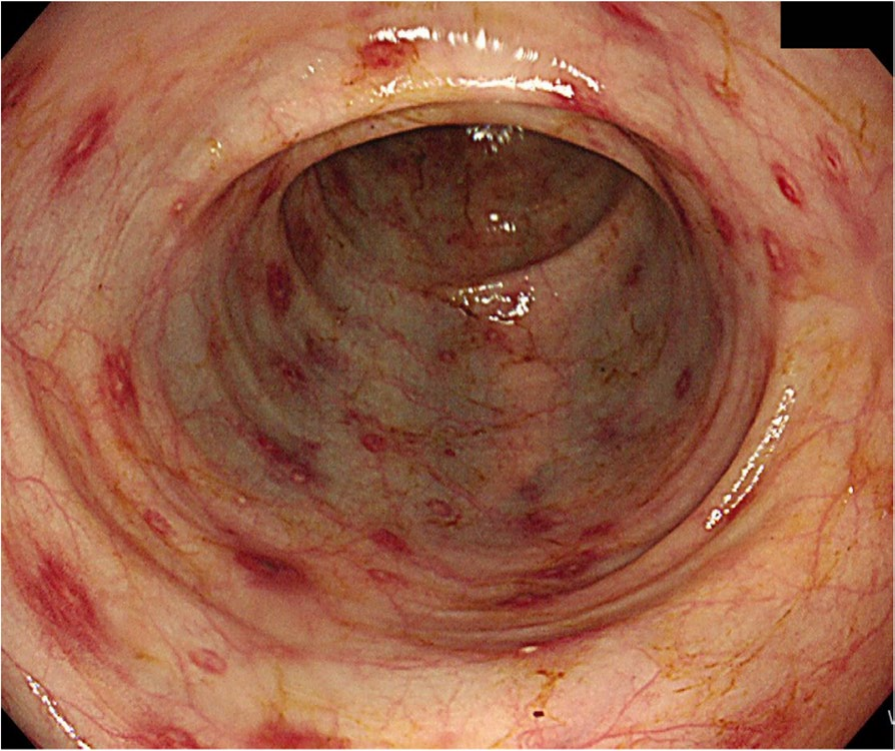
Endoscopic image shows multiple mucosal erythema, petechiae, and ulcers

The serum IgA level was 575 mg/dL (elevated, normal: 70–400 mg/dL) and complement component 3 and 4 levels were 35 mg/dL (decreased, normal: 90–180 mg/dL) and 8 mg/dL (decreased, normal: 10–40 mg/dL), respectively. Suspecting IgA vasculitis, we performed a punch biopsy of the skin. Microscopic examination revealed fibrinoid necrosis of vessels with extravasation of erythrocytes and neutrophils, suggesting leukocytoclastic vasculitis [Fig. 2]. On colonoscopy, multiple mucosal erythema, petechiae, and ulcers were observed throughout the colon, which indicated IgA vasculitis with gastrointestinal involvement [Fig. 3]. Microscopic findings of the colon biopsy showed ulceration with karyorrhectic debris, neutrophilic infiltration, and fibrin thrombi in lamina propria. Concerned about worsening MRSA bacteremia, we started low dose prednisolone therapy (0.5 mg/kg/day, gradual tapering) for relieving IgA-vasculitis-related symptoms like lower leg purpura and abdominal pain.

**Fig. 4:**
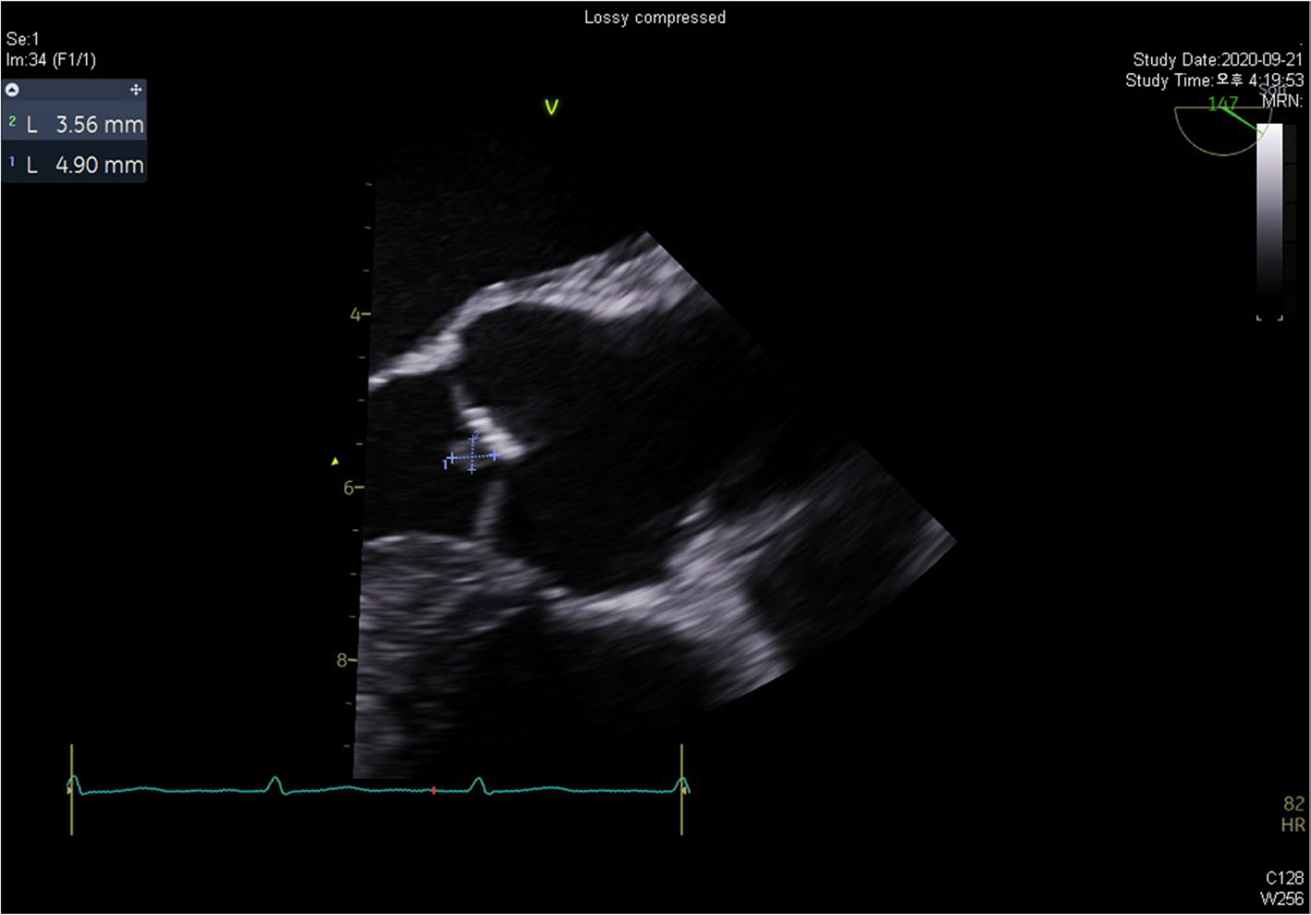
Transesophageal echocardiography image shows aortic valve vegetation

Although the patient was adequately treated with antibiotics, including intravenous vancomycin, fever occurred, and blood culture persistently yielded MRSA. Considering metastatic infection, a bone scan was performed, which did not reveal any evidence of bone infection or inflammation. However, transesophageal echocardiography (Vivid E95, GE Vingmed, 2020, Norway) revealed a 5 × 4 mm-sized, round, isoechoic, homogenous, mobile mass attached to the aortic valve [Fig. 4]. Based on these findings and the patient’s clinical features, we diagnosed him with infective endocarditis accompanied by IgA vasculitis and *C. difficile* infection. Because MRSA bacteremia was repeatedly detected on blood culture, we administered daptomycin in addition to vancomycin. Subsequently, fever, diarrhea, lower back pain, and lower extremity purpura improved, and MRSA bacteremia disappeared.

## Discussion and limitations

IgA vasculitis is diagnosed based on clinical manifestations, including skin, gastrointestinal, joint, and kidney involvement [1]. In our case, we diagnosed the patient with IgA vasculitis based on 2010 EULAR/PRINTO/PRES criteria [11]. Our patient had symmetric, palpable purpura in both lower extremities. Skin biopsy finding reveals leukocytoclastic vasculitis, characteristic of IgA vasculitis. Our patient experienced abdominal pain and diarrhea, which can be occurred by *C. difficile* infection and IgA vasculitis gastrointestinal involvement. However, colonoscopy findings suggesting vasculitis associated colitis rather than pseudomembranous colitis. Arthralgia/arthritis in IgA vasculitis usually involves the knees and ankles, symmetrically [2]. Our patient had lower back involvement, which is not typical of IgA vasculitis. We could not determine whether our patient had renal involvement because he had ESRD with anuric state since 2 years.

Leukocytoclastic vasculitis is nonspecific histopathologic finding. It can be found in several diseases including infective endocarditis and autoimmune diseases. Skin biopsy of immunofluorescence with IgA deposition is helpful for more precise IgA vasculitis diagnosis. A lack of immunofluorescence study is our study’s limitation. However, as mentioned above, IgA vasculitis is mainly a clinical diagnosis with peculiar skin purpura. We diagnosed him with IgA vasculitis based on clinical features compatible with IgA vasculitis. Serologic tests including IgA, complement components, negative for ANA, ANCA and normal eosinophil count can support for rule out other autoimmune diseases.

IgA vasculitis rarely occurs in ESRD patients undergoing hemodialysis. Since Esposito et al. first described it in 1999, only a few cases have been reported; we have summarized them in Table 1. In previous cases, diagnosis was based on clinical manifestations and skin biopsy findings. Gao et al. (2014) reported a case of IgA vasculitis triggered by catheter-related infection with *Staphylococcus aureus* bacteremia in a patient undergoing chronic hemodialysis [7]. Other reports did not describe the etiology of IgA vasculitis. IgA vasculitis is an immune-mediated vasculitis involving small vessels. It is unknown why it rarely occurs in patients undergoing hemodialysis; we hypothesize that it is related to immune system alteration in ESRD. Chronic uremic milieu is known to affect both the innate and adaptive immune systems [12]. Immune dysfunction is associated with defective CD86 expression in monocytes, leading to B7/28 pathway malfunction and impaired antigen-presenting function [13]. Changes in costimulatory molecules (CD80 and CD86) on the dendritic cell surface reduce antigen-presenting ability [14]. Moreover, toll-like receptor 4 (TLR4) function is diminished in patients undergoing hemodialysis and diminished TLR4 function leads to reduced production of cytokines [15]. These immune system changes might poorly respond to several antigens in ESRD and could be the cause of lower incidence in IgA vasculitis in patients undergoing hemodialysis. However, further studies on this topic are needed.

**Table 1:** Summary of previous reports of IgA vasculitis in patients with ESRD

Age/Sex	Cause of ESRD	Clinical manifestations	Laboratory findings	Treatment outcome	Year (reference number)
50/M	DM (biopsy proven)	Purpura Hematuria Abdominal pain Arthralgia	Normal C3, C4 Normal IgA	Resolution after steroid administration	2007 [6]
69/M	DM	Purpura Abdominal pain Hematuria	Elevated IgA, IgG Low C3 p-ANCA (+)	Resolution after antibiotic and steroid administration	2014 [7]
63/M	HTN	Purpura Abdominal pain Vomiting Melena Arthralgia	Low C3, C4	Died of sepsis	2019 [8]
61/M	DM	Purpura Abdominal pain Vomiting, Diarrhea	Low C3	Resolution after steroid administration	2020 [9]
70/M*	DM	Purpura Abdominal pain Diarrhea Arthralgia	Elevated IgA Low C3 Normal C4	Resolution after antibiotic and steroid administration	2021

*The Present case

*ESRD*: end stage renal disease; *M*: male; *DM*: diabetes mellitus; *HTN*: hypertension; *C3*: complement component 3; *C4*: complement component 4; *IgA*: immunoglobulin A; *IgG*: immunoglobulin G; *p-ANCA*: perinuclear anti-neutrophil cytoplasmic antibody

The etiology of IgA vasculitis remains unknown. It occurs in genetically susceptible individuals and is triggered by environmental factors such as infection or medications [16]. Here, infective endocarditis with MRSA bacteremia caused IgA vasculitis. The patient had hospitalized with AV graft infection 1 month ago, infective endocarditis could be intercurrent infection masked by antibiotic use. Also, infective endocarditis could be induced by AV graft infection consequently. However, during previous admission, the infected AV graft had been surgically removed with enough duration of antibiotic use. When the patient had discharged, he had any other symptom of infective endocarditis including fever. Although mucosal infection is the major cause of IgA vasculitis, several cases of IgA vasculitis with infective endocarditis have been reported. More than half of the patients reported as IgA-vasculitis-related infective endocarditis had risk factors for infective endocarditis such as intravenous drug use, history of infective endocarditis, congenital heart disease and hepatitis C infection [10, 17–20]. Hemodialysis is also a risk factor for infective endocarditis because of recurrent venipuncture. Therefore, infective endocarditis should be considered when purpura is noticed in patients undergoing hemodialysis. A few cases of IgA vasculitis triggered by *C. difficile* infection have been reported [21]. However, here, colonoscopy revealed gastrointestinal tract involvement. We therefore considered *C. difficile* infection as an independent complication due to previous antibiotic use which was administered 1 month ago.

Because IgA vasculitis recovers spontaneously, its treatment is often symptomatic. In cases with severe gastrointestinal and renal impairment, steroid treatment is required. Corticosteroids relieve arthralgia/arthritis and abdominal pain but are not effective against skin purpura in IgA vasculitis [3, 4]. Our patient was not initially administered steroids because of the risk of aggravation of bacterial infection and infective endocarditis. But lower leg purpura, gastrointestinal and joint symptoms aggravated which could not be correlated with infection course. This clinical course could not be explained by infective endocarditis alone. We administered low-dose steroids with closed monitoring of bacterial infection, and his symptoms improved.

In conclusion, we report the first case of IgA vasculitis triggered by infective endocarditis in a Korean patient undergoing hemodialysis. Patients undergoing hemodialysis are at a high risk of infection because of immune dysfunction and frequent venipuncture. The incidence of infective endocarditis associated with IgA vasculitis is very low, but it has been repeatedly reported. Therefore, it is necessary to consider infective endocarditis in patients with clinical features that indicate IgA vasculitis.

## Data Availability

All generated data were obtained from Veterans Health Service Medical Center and they are included in the published article.

## Abbreviations

C.difficile: *Clostridium difficile*
C3: Complement component 3
C4: Complement component 4
DM: Diabetes mellitus
ESRD: End-stage renal disease
HTN: Hypertension
IgA: Immunoglobulin A
IgG: Immunoglobulin G
MRSA: Methicillin-resistant *Staphylococcus aureus*
p-ANCA: Perinuclear anti-neutrophil cytoplasmic antibody
TLR4: Toll-like receptor 4
